# Comparison of genome-wide gene expression in suture- and alkali burn-induced murine corneal neovascularization

**Published:** 2011-09-02

**Authors:** Changkai Jia, Wei Zhu, Shengwei Ren, Haijie Xi, Siyuan Li, Yiqiang Wang

**Affiliations:** Shandong Provincial Key Lab of Ophthalmology, Shandong Eye Institute, Shandong Academy of Medical Sciences, Qingdao, China

## Abstract

**Purpose:**

Suture placement and alkali burn to the cornea are often used to induce inflammatory corneal neovascularization (CorNV) models in animals. This study compares the changes in genome-wide gene expression under these two CorNV conditions in mice.

**Methods:**

CorNV were induced in Balb/c mice by three interrupted 10–0 sutures placed at sites about 1 mm from the corneal apex, or by alkali burns that were 2 mm in size in the central area of the cornea. At the points in time when neovascularization progressed most quickly, some eyeballs were subjected to histological staining to examine CorNV and inflammatory cells infiltration, and some corneas were harvested to extract mRNA for microarray assay. After normalization and filtering, the microarray data were subject to statistical analysis using Significance Analysis of Microarray software, and interested genes were annotated using the Database for Annotation, Visualization, and Integrated Discovery (DAVID) program. The expression change of classical proangiogenic molecule like vascular endothelial growth factor (VEGF) and antiangiogenic molecule like pigment epithelium-derived factor (PEDF) was further verified using western blotting.

**Results:**

Suture placement induced CorNV in the areas between the suture and limbus, but did not affect the transparency of the yet unvasuclarized areas of the corneas. In contrast, alkali burn caused edema and total loss of transparency of the whole cornea. Histology showed that sutures only caused localized epithelial loss and inflammatory infiltration between the suture and limbus, but chemical burn depleted the whole epithelial layer of the central cornea and caused heavy cellular infiltration of the whole cornea. At day 5 after suture placement, 1,055 differentially expressed probes were identified, out of which 586 probes were upregulated and 469 probes were downregulated. At a comparable time point, namely on day 6 after the alkali burn to the corneas, 472 probes were upregulated and 389 probes were downregulated. Among these differentially expressed probes, a significant portion (530 probes in total, including 286 upregulated and 244 downregulated probes) showed a similar pattern of change in both models. Annotation (using DAVID) of the overlapping differential genes revealed that the significant enrichment gene ontology terms were “chemotaxis” and “immune response” for the upregulated genes, and “oxidation reduction” and “programmed cell death” for the downregulated genes. Some genes or gene families (e.g., S100A family or α-, β-, or γ-crystallin family) that had not been related to corneal pathogenesis or neovascularization were also revealed to be involved in CorNV. VEGF was upregulated and PEDF was stable as shown with western blotting.

**Conclusions:**

Sutures and alkali burn to the corneas produced types of damage that affected transparency differentially, but gene profiling revealed similar patterns of changes in gene expression in these two CorNV models. Further studies of the primary genes found to be involved in CorNV will supplement current understanding about the pathogenesis of neovascularization diseases.

## Introduction

Neovascularization, referring to the growth of abnormal vessels, is caused by the disruption of the balance between proangiogenic and antiangiogenic molecules [1-3]. It is a common pathological process observed in tumor growth and metastases, rheumatoid disease, and corneal and retinal disorders. Generally, the most intensively studied proangiogenic molecules include vascular endothelial growth factor (VEGF), basic fibroblast growth factor (bFGF), and interleukin-8 (IL-8), as well as the antiangiogenic molecules including angiostatin, endostatin, pigment epithelium–derived factor (PEDF), and so on. Specifically in cornea, the avascularity of corneas is a necessity for corneal transparency and relies on some properties of this tissue [4-7], like the expression of soluble VEGF receptor [8]. Some disorders, such as infections, degeneration, graft rejection, misuse of contact lenses, and chemical or physical damage, all can lead to loss of balance and can induce corneal neovascularization (CorNV). Though CorNV is a programmed response aimed at recovering homeostasis in insulted corneas, CorNV impairs vision. Thus, its prevention or correction is needed in most cases. The mechanisms of CorNV are sophisticated and are not clearly understood yet, and many studies on CorNV at the molecular level are based on knowledge about neovascularization in other tissues or in other pathological processes. To create a picture of gene expression at the genomic scale during the development of CorNV, microarray was used in two popular experimental CorNV models, namely suture placement and chemical burn induced CorNV in mice (S-CorNV and CB-CorNV). These two artificial etiological factors are believed to produce inflammatory process associated with CorNV pathogenesis after insults to cornea, like trauma, dry eye, chemical burn, etc. Microarray technology was chosen because of its strength in monitoring the expression of thousands of genes in a high-throughout manner, as well as in a quantitative manner. We expected that, besides uncovering the behavior of those conventional proangiogenic or antiangiogenic factors in CorNV, this study would also reveal some genes that had not been related to CorNV, thus providing new clues to understanding the pathogenesis of CorNV.

## Methods

### Animals

Balb/c mice, 6–8 weeks old, were used in this research. All mice were purchased from Beijing Pharmacology Institute, Chinese Academy of Medical Sciences (Beijing, China). Use of animals was approved by institution and observed the ARVO Statement for the Use of Animals in Ophthalmic and Vision Research.

### Corneal neovascularization models

Mice were anesthetized with ketamine (50 mg/kg) and chlorpromazine hydrochloride (10 mg/kg) by intraperitoneal injection. Compound tropicamide eye drops (Santen, Osaka, Japan) and 0.5% proparacaine hydrochloride (Alcon-Couvreur, Puurs, Belgium) were applied topically for corneal anesthesia. CorNV was induced by suture placement or chemical burn, as previously described by others [9]. Briefly, a corneal trephine 2 mm in diameter was pressed lightly on the central cornea to make a circular mark. Three interrupted 10–0 polypropylene sutures (MANI Inc., Togichi, Japan) spanning the mark were placed through the epithelial and stromal layers, but without penetrating the endothelial layer. To cause alkali burn to the cornea, a piece of filter paper (2.0 mm in diameter) soaked with 2 μl of 1 mol/l NaOH solution was placed on the central corneal surface for 40 s, followed by immediate rinsing with 30 ml of 0.9% saline buffer.

### Histology

Mice eyeballs were formalin ﬁxed, paraffin embedded, and sectioned at a thickness of 4 μm for routine histological processing. After staining with hematoxylin and eosin, vessels and cell infiltration were examined by light microscopy.

### Isolation of total RNA and microarray procedure

The procedure for total RNA isolation and microarray assay was described earlier [10]. In brief, at the desired time points after CorNV induction, corneas were excised using a 2 mm diameter trephine and placed in ice-cold TRIzol reagent (Invitrogen, Gaithersburg, MD), with five corneas from each model pooled into one sample. The untreated corneas from the same mice were used as controls. Three pairs of samples were prepared for each model. Total RNA was extracted using isopropanol precipitation and was purified using NucleoSpin RNA clean-up columns (Macherey-Nagel, Düren, Germany). Dual cRNA labeling and microarray hybridizations were performed by Capital Bio Corporation using Capital Bio cRNA labeling kits and the Capital Bio 36 K Mouse Genome Oligo Array (Capital Bio, Beijing, China) [11,12]. The array comprises 35,852 70-mer oligonucleotide probes representing approximately 25,000 genes of Mouse Genome Version 4.0 (Operon Biotechnologies, Huntsville, AL). Three replicate arrays were used for each model. After hybridization, the arrays were scanned using a LuxScan 10KA (Capital Bio), and signals were processed with LuxScan 3.0 software (Capital Bio). Intra-array normalization was done using the LOcally Weighted Scatter plot Smoothing (LOWESS) linearization method. Inter-array normalization of the whole data set was performed according to the global means of Cy5 and Cy3 signals [12].

### Microarray data analysis

In each array, the probes that passed various quality check and gave signal intensity of over 1,500 were labeled as Expressed, otherwise a probe was considered Marginal (intensity 800–1500) or Absent (less than 800). Only those probes that were expressed in at least two out of three chips were taken into account for further analysis. After log^2^ transformation of the fold values, one class *t*-test analysis was performed by Significance Analysis of Microarray software SAM 3.0 (Stanford University, Stanford, CA) [13] to determine their change or stability in the CorNV group compared with the control group. Those genes that gave less than a 1% false discovery rate (FDR) and no less than twofold changes were considered to be differentially expressed genes. Last, the differentially expressed genes identified in each CorNV model were compared and annotated using the Database for Annotation, Visualization and Integrated Discovery (DAVID, v6.7) with the whole murine genome as the background [14]. Gene Ontology (GO) categorization was performed using a modified Fisher exact test, and the p value for each GO category was calculated as a expression analysis systematic explorer (EASE) score [15]. An EASE score of not more than 0.01 indicated a significant enrichment. Hierarchical clusters were performed for different sets of interested genes using the Cluster 3.0 program with the Pearson correlation (uncentered) distance, average linkage [16,17], and the resulting CDT files transferred into heat maps using Java Treeview [18]. The complete sets of normalized data of this microarray assay are deposited in the NCBI Gene Expression Omnibus (GEO) with a GEO accession number GSE23347.

### Antibodies and western blotting

Corneas were harvested by cutting along the centric side of limbal line and placed in RIPA lysis buffer (Beyotime, Shanghai, China) for total protein preparation. For each sample, two corneas were combined and 100 μl buffer were used. The tissues was cut with scissors into small pieces and homogenized using a tissue tearor (Biospec Products, Inc., Bartlesville, OK). After spinning at 11,500× g for 10 min, 80 μl cleared lysate were mixed with 20 μl 5× loading buffer. After boiling at 95 °C for 5 min, 10 μl samples were resolved on 12% SDS–PAGE gel and then transferred to a polyvinylidene difluoride (PVDF) membrane (Millipore, Billerica, MA). The blots were blocked in 5% non-fat dry milk dissolved in TBST (20 mM Tris PH7.5, 0.5 mM NaCl, 0.05% Tween-20) for 1 h and then incubated with the primary antibody in TBST for 2 h, followed by incubation with HRP-conjugated secondary antibody for 1 h. All incubations were done at room temperature and three washes with 10 ml TBST were applied between each step. Primary antibodies include anti-mouse PEDF antibody (sc-25594; Santa Cruz Biotechnology Inc., Santa Cruz, CA), anti-VEGF antibody (ab-3109; Abcam Biotechnology, Cambridge, MA). Secondary antibodies include Peroxidase-Conjugated AffiniPure Goat Anti-Rabbit IgG (H^+^L; ZB-2301; Zhongshan Golden Bridge, Beijing, China) and Peroxidase-Conjugated AffiniPure Anti-Mouse IgG (ZB-2305, Zhongshan Golden Bridge).The blot was developed with SuperSignal West Pico (NCI5079,Thermo Fisher Scientific, Rockford, USA) and exposed to X-ray film (Kodak, Rochester, NY). For detecting GAPDH, the PVDF membrane was regenerated using Stripping Solution (Applygen Technologies Inc., Beijing, China) and detected using an GAPDH detection kit (KC-5J5,KangChen Biotechnology, Shanghai, China) as suggested by the manufacturer.

## Results and Discussion

### Corneal neovascularization in two models

**Figure 1:**
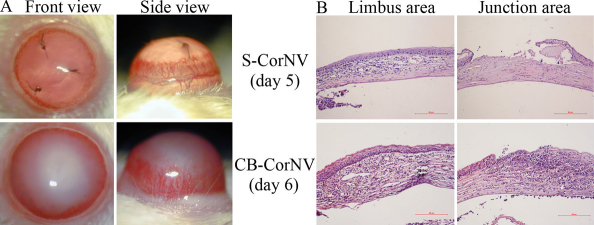
Gross and histology presentations of corneas of corneal neovascularization models. **A**: Front views and side views of corneas with corneal neovascularization under slit lamp. **B**: Hematoxylin-eosin staining of corneas at limbus area and junction area, the latter of which refers to the area of the suture stitch in the S-corneal neovascularization model and the margin of direct chemical burn. The red scale bar represent 50 μm. Please note the difference of manifestations in these two models, especially in terms of transparency of the corneas.

Both S-CorNV and CB-CorNV have been used successfully, including in our laboratory [10,19], when studying either mechanisms of CorNV development or interference from exogenous factors in the development of CorNV. The conclusions drawn using one model are presumed to be applicable to all CorNV. We previously used both models without any specific preference; by contrast, this study tried to define any commonalities or differences between these two models. Clinically, suture placement did not cause other significant changes to corneal transparency except for the development of neovascularization, but chemical burn caused total loss of transparency of the burned area, starting from the burn (Figure 1A). Later, the haze extended from the edge of the burn to the limbus. The difference in the gross appearance of corneas in these two models correlated with the histological changes (Figure 1B). While the suture did not affect the intactness of the epithelial layer in areas other than the suture punch, the chemical burn depleted the entire epithelial layer of the central corneas. Correspondingly, the suture caused inflammatory infiltration limited to the area between the suture and limbus, but the chemical burn caused heavy infiltration and edema of the whole cornea. In spite of the significant gross difference in these two models, the appearance and progress of CorNV in chemically burned corneas was slightly slower than that in sutured corneas. S-CorNV progressed most quickly at day 5, and CB-CorNV at day 6 (Figure 1), reaching maximal length at day 10 and day 14, respectively (data not shown). This paper focused on data obtained at the fast-growth time points, namely day 5 for the suture and day 6 for the chemical burn, respectively.

### Comparison of differentially expressed genes in two corneal neovascularization models

**Figure 2:**
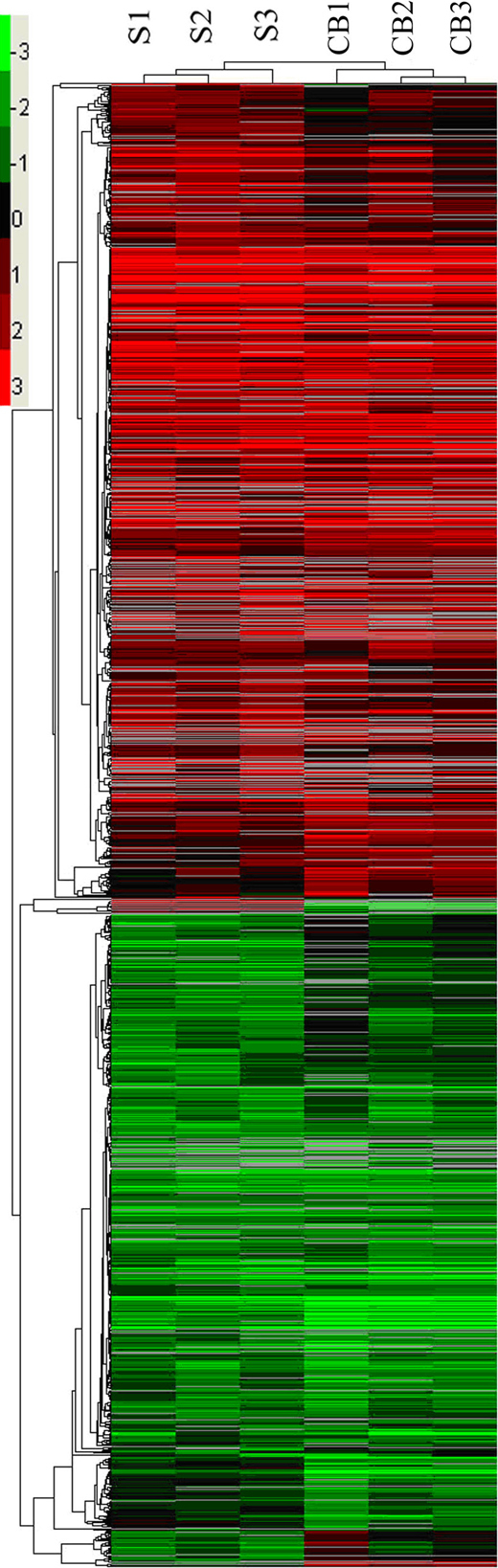
Hierarchical clustering of all 1,386 differentially expressed probes in either the S- or the CB-corneal neovascularization model. The color bar located in upper left corner stands for the folds of probe changes (in log_2_ value), while the gray color in the heat maps indicates that the value was absent in a specified microarray. S, suture; CB, chemical burn.

Merely by checking the gross presentation or histology of corneas in these two conditions as described above, we expected CB-CorNV to affect the expression of more genes or to affect them to a greater extent than S-CorNV would. However, gene profiling using microarray showed that S-CorNV affected gene expression more than did CB-CorNV. Among all the 35,872 probes (excluding various controls) in this chip, 7,138 that passed the filter criteria were identified as being expressed in S-CorNV, and 7,109 in CB-CorNV, for a total of 7,766 probes expressed in at least one model. Namely, about 92% of all expressed probes were detected in both models, reflecting the consistency and reliability of the data sets. Statistical analysis using SAM software, with the thresholds set at FDR<1% and fold change≥2, revealed that 1,055 probes (accounting for 14.78% of all expressed genes in the S-CorNV model) were differently expressed. Among them, 586 probes were upregulated and 469 probes were downregulated. Similarly, in the CB-CorNV model, 472 probes were upregulated and 389 were downregulated, making a total of 861 probes (12.11%) differentially expressed. Among these differentially expressed genes, 530 probes in total overlapped in the two models, including 286 upregulated and 244 downregulated probes (Table 1). Some probes remained unchanged in one model but up- or downregulated in the other model. No probes manifested contradictory changes in the two models; namely, no probes were upregulated in one model while being downregulated in the other model. To allow a better overview of the changes of all 1,386 changed probes, hierarchical clustering analysis was performed, and is shown in Figure 2.

**Table 1:** Grouping of probes expressed in at least one corneal neovascularization model.

** **	**S-CorNV**			
**CB-CorNV**	**Up-Reg**	**Unchanged**	**Down-Reg**	**Absent**
Up-Reg	G1: 286	G4: 99	0	G9: 87
Unchanged	G2: 203	0	G6: 186	0
Down-Reg	0	G5: 114	G7: 244	G10: 31
Absent	G3: 97	0	G8: 39	0

In each individual microarray, probes were identified as E (expressed), M (marginal), or A (absent) in an individual array after linearization and normalization as described in text, and only those classified as E were calculated for ratio values of the signal intensities of experimental samples against controls in the same arrays. For statistical purposes, probes marked as E in only one of three microarrays in one model were classified as A in this table. Down-Reg meant a signal intensity ratio of the experimental sample/control ≤0.5 plus FDR<1%, and Up-Reg meant ≥2.0 plus FDR<1%. Unchanged was for those of 0.5<ratio<2 or FDR>1%.

### Complimentary validation of expression patterns of *VEGFA* and *PEDF*

**Figure 3:**
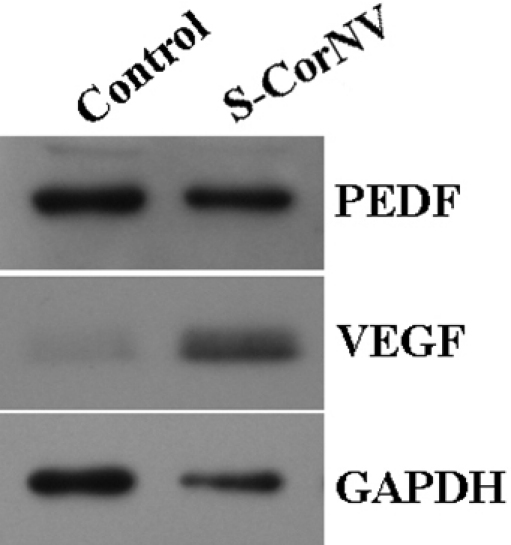
Change of expression of PEDF and VEGF detected by western blot in corneal neovascularization. Samples were harvested at day 5 after S-CorNV induction and proteins of equivalent to one fifth cornea were loaded and detected by western blot. Please note that due to differential levels of two factors in samples, the exposing time of blotted membrane against X-films varied, namely about 45 s for PEDF, 2 min for GAPDH, and about 1 h for VEGF. Shown was one representative of three experiments that gave similar conclusions.

It was interesting and unexpected to note that such classical angiogenesis modulators as *VEGFA* and *PEDF* were not among the aforementioned 1,055 genes that produced significant alterations at mRNA levels in either CorNV model. Studying the raw array data showed that *VEGFA* signals fell below the threshold set by analysis criteria thus regarded Absent (namely, unexpressed) in the corneas. For example, four probes with Oligo ID being M400000704, M400000705, M400000706, and M400000707, respectively, are included in the array for the *VEGFA* gene. The signals for these four probes in the S-CorNV group shown as “experimental sample signal/control sample signal” were 272.3±30.7/56±52.5 (mean±standard deviation for three arrays), 412.3±80.0/220.6±74.7, 159.6±43.5/31.3±161.8, and 238.3±79.1/97.6±88.8, respectively. If the above filtering threshold would be arbitrarily disregarded and the fold changes provided by the automatic analysis accompanied with the array signal results, these four probes would produce a ratio of 2.7 for *VEGFA*, namely *VEGFA* mRNA would be regarded upregulated by 2.7 fold in S-CorNV compared with control corneas. On the contrary, *PEDF* gave high enough signals (between 4,000 and 6,300, not shown) and were classified as “expressed” in all samples, the change in S-CorNV was not significant (the experiment/control ratios in three arrays were 0.754, 0.890, and 1.076, respectively). To double check the expression levels of these two genes, western blot was performed and confirmed that PEDF was expressed at high level in normal control corneas but did not change significantly in S-CorNV, while VEGFA showed marginal expression but significantly upregulated in S-CorNV (Figure 3). The situation of *VEGFA* confirmed the rationale that microarray serves an effective primary screening method, and other complementary methods would be mandatory under some situation, like when certain highly suspected genes gave Marginal or Absent signals in microarray assay.

### Functional annotation of differentially expressed genes

The DAVID functional annotation tool was used to annotate genes in various groups (refer to Table 1), mainly based on the GO biological process. The enriched GO terms in each group are summarized in Table 2. While most of the jointly upregulated GO terms (G1 group) were associated with “cell migration,” “defense/immune/inflammation responses,” or “tissue development/organization/homeostasis,” the only two main GO terms enriched in the jointly downregulated group (G7 group) were “oxidation reduction” and “programmed cell death” (Table 2). Trying to reveal common pathways determining development of two apparently different CorNV models, the whole list of involved genes in several promising GO terms from Group 1 are also given. They include “chemotaxis” (Table 3), “immune response” (Table 4), “inflammatory response” (Table 5), and “regulation of cytokine production” (Table 6).

**Table 2:** Enriched gene ontology terms according to biologic process in various groups of genes.

**Enriched GO terms in each group**	**Count^a^**
**G1 group: upregulated in both S-CorNV and CB-CorNV**	
**Cell migration**	
chemotaxis (see Table 3)	18
leukocyte chemotaxis	9
leukocyte migration	10
locomotory behavior	18
cell migration	12
**Host defense responses**	
immune response (see Table 4)	32
defense response	29
inflammatory response (see Table 5)	21
response to wounding	25
acute inflammatory response	10
acute-phase response	6
response to organic substance	16
response to molecule of bacterial origin	5
antigen processing and presentation of exogenous antigen	6
immunoglobulin mediated immune response	7
B cell mediated immunity	7
lymphocyte mediated immunity	7
positive regulation of response to stimulus	10
regulation of cytokine production (see Table 6)	8
regulation of tumor necrosis factor production	4
response to oxidative stress	6
response to cytokine stimulus	4
positive regulation of acute inflammatory response	3
positive regulation of multicellular organismal process	8
cytokine-mediated signaling pathway	5
defense response to Gram-negative bacterium	3
**Tissue development or organization**	
eye development	10
lens development in camera-type eye	5
multicellular organismal homeostasis	6
extracellular structure organization	8
regulation of biomineral formation	4
regulation of bone mineralization	4
regulation of homeostatic process	6
cellular cation homeostasis	8
collagen fibril organization	4
homeostatic process	17
cellular homeostasis	12
vasculature development	10
epidermis development	7
sensory organ development	10
ectoderm development	7
epithelium development	10
epithelial cell differentiation	7
lens fiber cell differentiation	3
cellular iron ion homeostasis	4
positive regulation of developmental process	9
blood vessel development (refer to Table 7)	10
**Metabolism**	
intermediate filament-based process	4
icosanoid biosynthetic process	4
unsaturated fatty acid biosynthetic process	4
icosanoid metabolic process	4
**G2 group: upregulated in S-CorNV, unchanged in CB-CorNV**	
mitosis	8
nuclear division	8
organelle fission	8
M phase	9
cell cycle phase	9
**G3 group: upregulated in S-CorNV, absent in CB-CorNV**	
not any	
**G4 group: unchanged in S-CorNV, upregulated in CB-CorNV**	
chordate embryonic development	9
embryonic development ending in birth or egg hatching	9
endothelial cell morphogenesis	2
**G5 group: unchanged in S-CorNV, down-regulated in CB-CorNV**	
not any	
**G6 group: down-regulated in S-CorNV, unchanged in CB-CorNV**	
**Lipid metatolism**	
lipid biosynthetic process	15
steroid metabolic process	12
steroid biosynthetic process	8
cholesterol metabolic process	7
carboxylic acid biosynthetic process	8
organic acid biosynthetic process	8
fatty acid biosynthetic process	6
oxidation reduction	16
**Host response**	
cellular response to extracellular stimulus	5
cellular response to starvation	4
epithelium development	9
**Differentiation**	
epithelial cell differentiation	6
epidermis development	6
keratinocyte differentiation	4
epidermal cell differentiation	4
Wnt receptor signaling pathway	6
ectoderm development	6
regulation of neuron differentiation	5
**G7 group: down-regulated in both S-CorNV and CB-CorNV**	
oxidation reduction (see Table 9)	19
programmed cell death	14
retinol metabolic process	3
**G8 group: down-regulated in S-CorNV, absent in CB-CorNV**	
not any	
**G9 group: absent in S-CorNV, upregulated in CB-CorNV**	
response to organic substance	10
response to steroid hormone stimulus	4
immune response	8
oxidation reduction	9
icosanoid metabolic process	3
**G10 group: absent in S-CorNV, down-regulated in CB-CorNV**	
not any	

“Enrichment” means that a specific GO terms gives an Expression Analysis Systematic Explore (EASE) score less than 0.01. To simplify the presentation of the enriched terms, the GO terms that obviously duplicate others were not shown. For example, “cellular response to nutrient levels” in Group 6 was not shown due to its duplication to “cellular response to starvation.” Similarly, “Cell death,” “death,” and “apoptosis” in Group 7 were duplicates of “programmed cell death,” thus not shown either. ^a^Numbers of probes in the specific group or GO term.

**Table 3:** Genes associated with the gene ontology term “chemotaxis” in the upregulated gene list.

**Ref_Seq ID**	**Gene symbol**	**Gene name**	**S-CorNV folds**	**CB-CorNV folds**
NM_010185	*Fcer1g*	Fc receptor, IgE, high affinity I, gamma polypeptide	10.03	8.98
NM_010188	*Fcgr3*	Fc receptor, IgG, low affinity III	8.28	6.51
NM_013650	*S100a8*	S100 calcium binding protein A8 (calgranulin A)	11.50	9.08
NM_009114	*S100a9*	S100 calcium binding protein A9 (calgranulin B)	45.35	15.97
NM_011333	*Ccl2*	chemokine (C-C motif) ligand 2	48.21	10.59
NM_011124	*Ccl21a*	chemokine (C-C motif) ligand 21A; predicted gene 1987	4.97	5.78
NM_009139	*Ccl6*	chemokine (C-C motif) ligand 6	16.26	13.82
NM_021443	*Ccl8*	chemokine (C-C motif) ligand 8	12.86	7.47
NM_011338	*Ccl9*	chemokine (C-C motif) ligand 9	13.72	10.02
NM_009140	*Cxcl2*	chemokine (C-X-C motif) ligand 2	35.49	9.30
NM_203320	*Cxcl3*	chemokine (C-X-C motif) ligand 3	8.58	18.49
NM_009898	*Coro1a*	coronin, actin binding protein 1A	17.93	6.91
NM_008039	*Fprl1*	formyl peptide receptor 2	23.07	15.79
NM_008361	*Il1b*	interleukin 1 beta	22.61	9.88
NM_008489	*Lbp*	lipopolysaccharide binding protein	5.32	4.26
NM_019932	*Cxcl4*	platelet factor 4	6.36	15.72
NM_011335	*Ccl21b*	chemokine (C-C motif) ligand 21B	3.80	4.56
NM_023052	*Ccl21c*	chemokine (C-C motif) ligand 21C (leucine)		
NM_009141	*Cxcl5*	similar to LPS-induced CXC chemokine; chemokine (C-X-C motif) ligand 5	71.97	153.33

The values were the geometric mean of the ratios of signal intensity of experimental sample to normal control corneas.

**Table 4:** Genes associated with the gene ontology term “immune response” in the upregulated gene list.

**Ref_Seq ID**	**Gene symbol**	**Gene name**	**S-CorNV folds**	**CB-CorNV folds**
NM_010819	*Clecsf8*	C-type lectin domain family 4, member d	27.78	17.05
NM_020001	*Clecsf10*	C-type lectin domain family 4, member n	14.52	15.83
NM_009841	*Cd14*	CD14 antigen	25.69	20.90
NM_001042605	*Cd74*	CD74 antigen (invariant polypeptide of major histocompatibility complex, class II antigen-associated)	2.44	3.04
NM_010185	*Fcer1g*	Fc receptor, IgE, high affinity I, gamma polypeptide	10.03	8.98
NM_010188	*Fcgr3*	Fc receptor, IgG, low affinity III	8.28	6.51
NM_007388	*Acp5*	acid phosphatase 5, tartrate resistant	2.60	4.30
NM_011333	*Ccl2*	chemokine (C-C motif) ligand 2	48.21	10.59
NM_011124	*Ccl21a*	chemokine (C-C motif) ligand 21A; predicted gene 1987	4.97	5.78
NM_009139	*Ccl6*	chemokine (C-C motif) ligand 6	16.26	13.82
NM_021443	*Ccl8*	chemokine (C-C motif) ligand 8	12.86	7.47
NM_011338	*Ccl9*	chemokine (C-C motif) ligand 9	13.72	10.02
FNM_009915	*Ccr2*	chemokine (C-C motif) receptor 2	27.51	16.93
NM_008176	*Cxcl1*	chemokine (C-X-C motif) ligand 1	14.52	7.16
NM_019568	*Cxcl14*	chemokine (C-X-C motif) ligand 14	3.34	2.30
NM_009140	*Cxcl2*	chemokine (C-X-C motif) ligand 2	35.49	9.30
NM_203320	*Cxcl3*	chemokine (C-X-C motif) ligand 3	8.58	18.49
NM_007574	*C1qg*	complement component 1, q subcomponent, C chain	4.22	3.86
NM_007572	*C1qa*	complement component 1, q subcomponent, alpha polypeptide	5.50	7.72
NM_009777	*C1qb*	complement component 1, q subcomponent, beta polypeptide	5.22	5.85
NM_008147	*Lilrb4*	leukocyte immunoglobulin-like receptor, subfamily B, member 4	36.73	24.18
NM_010378	*H2-Aa*	histocompatibility 2, class II antigen A, alpha;	2.53	3.32
NM_010381	*H2-Ea*	histocompatibility 2, class II antigen E alpha	3.50	4.17
NM_207105	*H2-Ab1*	histocompatibility 2, class II antigen A, beta 1	4.38	4.84
NM_010382	*H2-Eb1*	histocompatibility 2, class II antigen E beta	3.56	4.51
NM_008361	*Il1b*	interleukin 1 beta	22.61	9.88
NM_153511	*Il1f9*	interleukin 1 family, member 9	2.20	2.83
NM_008489	*Lbp*	lipopolysaccharide binding protein	5.32	4.26
NM_019932	*Cxcl4*	platelet factor 4	6.36	15.72
NM_011335	*Ccl21b*	similar to beta chemokine Exodus-2; chemokine (C-C motif) ligand 21B;	3.80	4.56
NM_009760	*Bnip3*	predicted gene 14506; BCL2/adenovirus E1B interacting protein 3	3.76	3.11
NM_023785	*Cxcl7*	pro-platelet basic protein	61.39	32.60
NM_009141	*Cxcl5*	similar to LPS-induced CXC chemokine; chemokine (C-X-C motif) ligand 5	71.97	153.33

**Table 5:** Genes associated with the gene ontology term “inflammatory response” in the upregulated gene list.

**Ref_Seq ID**	**Gene symbol**	**Gene name**	**S-CorNV folds**	**CB-CorNV folds**
NM_010169	*F2r*	coagulation factor II (thrombin) receptor	2.97	3.30
NM_009252	*Serpina3n*	serine (or cysteine) peptidase inhibitor, clade A, member 3N	17.13	10.07
NM_009117	*Saa1*	serum amyloid A 1	26.09	9.43
NM_011315	*Saa3*	serum amyloid A 3	85.23	35.01
NM_133977	*Trf*	transferrin	3.41	2.73

Among all 21 genes in the GO terms of “inflammatory response,” the ones that overlapped with those in “immune response” were not shown, they included *Cd14*, *Fcgr3*, *Ccl2*, *Ccl21a*, *Ccl8*, *Ccr2*, *Cxcl1*, *Cxcl2*, *Cxcl3*, *C1qg*, *C1qa*, *C1qb*, *Il1b*, *Lbp*, *Ccl21b*/*Ccl21c*, and *Cxcl5*.

**Table 6:** Genes associated with the gene ontology term “regulation of cytokine production” in the upregulated gene list.

**Ref_Seq ID**	**Gene symbol**	**Gene name**	**S-CorNV folds**	**CB-CorNV folds**
NM_010169	*F2r*	coagulation factor II (thrombin) receptor	2.97	3.30
NM_010442	*Hmox1*	heme oxygenase (decycling) 1	6.21	4.82
NM_011157	*Prg*	serglycin	6.87	7.29

Among all 8 genes in the GO term of “regulation of cytokine production,” the ones that overlapped with those in “immune response” were not shown, they included *Cd14*, *Fcer1g*, *Fcgr3*, *Il1b*, and *Lbp*.

Even without going further into each gene’s function, it could be said that the genes enriched in these groups coincided the events described above (Table 2). Since blood vessel formation is the core component of neovascularization, we looked up all genes that belonged to the “blood vessel development” term (Table 7) among all changed probes. Surprisingly, none of the common proangiogenic or antiangiogenic factors, such as *VEGF* and *PEDF*, appear in this table. However, a group of lens crystallins included in the GO term of “structural constituent of eye lens,” which has recently been proven to be expressed physiologically in mammalian corneas [20], was among the upregulated probes (Table 8). Most of these lens crystallin genes were upregulated in S-CorNV at about 2–10 fold higher than in the CB-CorNV model. By contrast, the enzyme crystallins (e.g., *ALDH1A1* and *ALDH3A1*) which have long been proposed to be involved in antioxidation in ocular tissues [21-23], were significantly downregulated (Table 9). Among other genes that were listed as downregulated in the GO term of “oxidation reduction” (Table 9), the clustering of five members of the cytochrome P450 family in this group is quite suggestive, since this family has been proposed to be critical in angiogenesis, and inhibitors of them suppress angiogenesis [24].

**Table 7:** Genes associated with the gene ontology term “blood vessel development” that changed in either corneal neovascularization model.

**Ref_Seq ID**	**Gene symbol**	**Gene name**	**S-CorNV folds**	**CB-CorNV folds**
NM_198725	*Egfl7*	EGF-like domain 7	4.26	3.08
NM_010228	*Flt1*	FMS-like tyrosine kinase 1	2.01	2.31
NM_009930	*Col3a1*	collagen, type III, alpha 1	12.87	16.57
NM_015734	*Col5a1*	collagen, type V, alpha 1	2.99	3.12
NM_010442	*Hmox1*	heme oxygenase (decycling) 1	6.21	4.82
NM_008361	*Il1b*	interleukin 1 beta	22.61	9.88
NM_008610	*Mmp2*	matrix metallopeptidase 2	4.32	4.34
NM_007707	*Socs3*	suppressor of cytokine signaling 3	3.58	2.17
NM_009382	*Thy1*	thymus cell antigen 1, theta	3.57	2.68
NM_013749	*Tnfrsf12a*	tumor necrosis factor receptor superfamily, member 12a	4.08	4.81
NM_007709	*Cited1*	Cbp/p300-interacting transactivator with Glu/Asp-rich C-terminal domain 1	2.54	1.00
NM_053087	*Epgn*	epithelial mitogen	2.72	1.88
NM_009929	*Col18a1*	collagen, type XVIII, alpha 1	1.96	2.10
NM_030250	*Nus1*	nuclear undecaprenyl pyrophosphate synthase 1 homolog (S. cerevisiae)	1.97	2.04
NM_194054	*Rtn4*	reticulon 4	1.72	2.48
NM_007742	*Col1a1*	collagen, type I, alpha 1		3.93
NM_007950	*Ereg*	epiregulin		2.40
NM_009769	*Klf5*	Kruppel-like factor 5	0.36	1.40
NM_008943	*Psen1*	presenilin 1	0.42	0.67
NM_009154	*Sema5a*	semaphorin, 5A	0.42	0.45
NM_016907	*Spint1*	serine protease inhibitor, Kunitz type 1	0.49	0.84
NM_009373	*Tgm2*	transglutaminase 2, C polypeptide	0.27	0.21
NM_010197	*Fgf1*	fibroblast growth factor 1	0.66	0.29
NM_023517	*Tnfsf13*	tumor necrosis factor (ligand) superfamily, member 13	0.70	0.49
NM_026924	*OVOL2*	ovo-like 2 (Drosophila)		0.43

**Table 8:** Genes associated with the gene ontology term “structural constituent of eye lens” according to “molecular function” in upregulated genes.

**Ref_Seq ID**	**Gene symbol**	**Gene name**	**S-CorNV folds**	**CB-CorNV folds**
NM_013501	*Cryaa*	crystallin, alpha A	18.90	6.31
NM_009965	*Cryba1*	crystallin, beta A1	9.07	4.79
NM_021541	*Cryba2*	crystallin, beta A2	17.96	4.06
NM_023695	*Crybb1*	crystallin, beta B1	20.01	4.76
NM_007773	*Crybb2*	crystallin, beta B2	16.68	6.51
NM_021352	*Crybb3*	crystallin, beta B3	10.81	3.04
NM_144761	*Crygb*	crystallin, gamma B	12.38	4.36
NM_007775	*Crygc*	crystallin, gamma C	39.09	4.02
NM_007776	*Crygd*	crystallin, gamma D	16.12	4.61
NM_007777	*Cryge*	crystallin, gamma E	14.08	3.70
NM_027010	*Crygf*	crystallin, gamma F	14.93	4.78
NM_009967	*Crygs*	crystallin, gamma S	29.49	6.45

**Table 9:** Gene list assocated with the gene ontology term “oxidation reduction” in downregulated genes.

**Ref_Seq ID**	**Gene symbol**	**Gene name**	**S-CorNV folds**	**CB-CorNV folds**
NM_028133	*Egln3*	EGL nine homolog 3 (C. elegans)	0.47	0.36
NM_008706	*Nqo1*	NAD(P)H dehydrogenase, quinone 1	0.26	0.33
NM_007409	*Adh1*	alcohol dehydrogenase 1 (class I)	0.12	0.17
NM_009626	*Adh7*	alcohol dehydrogenase 7 (class IV)	0.11	0.40
XM_974140	*Aldh3b2*	alcohol dehydrogenase 3, member b2	0.34	0.38
NM_013467	*Aldh1a1*	aldehyde dehydrogenase family 1, subfamily A1	0.35	0.25
NM_007436	*Aldh3a1*	aldehyde dehydrogenase family 3, subfamily A1	0.24	0.13
NM_013777	*Akr1c12*	aldo-keto reductase family 1, member C12	0.32	0.16
NM_013778	*Akr1c13*	aldo-keto reductase family 1, member C13	0.32	0.16
NM_023066	*Asph*	aspartate-beta-hydroxylase	0.23	0.26
NM_007819	*Cyp3a13*	cytochrome P450, family 3, subfamily a, polypeptide 13	0.44	0.29
NM_018887	*Cyp39a1*	cytochrome P450, family 39, subfamily a, polypeptide 1	0.19	0.31
NM_172306	*Cyp4a12*	cytochrome P450, family 4, subfamily a, polypeptide 12B	0.11	0.39
NM_177406	*Cyp4a12*	cytochrome P450, family 4, subfamily a, polypeptide 12a	0.23	0.49
NM_025968	*Ptgr1*	prostaglandin reductase 1	0.25	0.14
NM_028725	*Sdr42e1*	short chain dehydrogenase/reductase family 42E, member 1	0.30	0.21
NM_201640	*Cyp4a31*	cytochrome P450, family 4, subfamily a, polypeptide 10	0.14	0.41
NM_007453	*Prdx6*	similar to Peroxiredoxin-6	0.30	0.38
NM_028454	*Tm7sf2*	transmembrane 7 superfamily member 2	0.34	0.41

Table 10 further lists some genes that were significantly changed during CorNV and deserve further studies. For example, since little information about the gene Corneal Endothelial-specific Protein-1 (NM_026358) is available and its function remains unclear [25], the fact that it was downregulated in both models suggested that this gene might be functionally involved in CorNV or that corneal endothelial cells might be also involved in CorNV development. Besides the genes of same family and that showed concerted changes (e.g., serine peptidase inhibitors), those genes that belong to same family but manifested opposite changes in one or two CorNV models also deserve attention. Detailed comparison of each member of such families will help to dissect the modulation of CorNV development. For instance, while *Col3a1* and *Col5a2* expression increased and *Col4a4* expression decreased in both models, change of keratin 12 was also opposite to that of other genes in its family. Opposite changes were also observed for interferon-induced transmembrane protein 3 and other interferon-induced proteins. Some hypothetical genes that have not been confirmed for any biologic functions by experimental study are also listed for comparison.

**Table 10:** Genes of specific interest.

**Ref_Seq ID**	**Gene symbol**	**Gene name**	**S-CorNV folds**	**CB-CorNV folds**
**Corneal endothelial specific protein**				
NM_026358	*CESP-1*	Corneal endothelial-specific protein 1	0.25±0.04	0.07±0.00
**Collagens**				
NM_009930	*Col3a1*	collagen, type III, alpha 1	12.94±1.69	16.90±4.05
NM_007735	*Col4a4*	collagen, type IV, alpha 4	0.31±0.07	0.22±0.08
NM_007737	*Col5a2*	collagen, type V, alpha 2	5.16±3.19	3.08±0.37
**Interferon induced proteins**				
NM_194069	*Ifi27l1*	interferon, alpha-inducible protein 27 like 1	0.41±0.04	0.39±0.08
NM_027320	*Ifi35*	interferon-induced protein 35	0.40±0.07	0.49±0.13
NM_133871	*Ifi44*	interferon-induced protein 44	0.43±0.04	0.42±0.07
NM_008331	*Ifit1*	interferon-induced protein with tetratricopeptide repeats 1	0.44±0.07	0.37±0.09
NM_025378	*Ifitm3*	interferon induced transmembrane protein 3	2.80±0.14	4.52±0.04
**Keratocans**				
NM_008438	*KERA*	keratocan	0.24±0.06	0.20±0.03
**Keratins**				
NM_010662	*Krt1–13*	keratin 13	3.25±0.44	3.23±1.58
NM_016958	*Krt1–14*	keratin 14	5.80±2.16	5.08±2.95
NM_008470	*Krt1–16*	keratin 16	14.16±3.28	6.09±0.69
NM_010663	*Krt1–17*	keratin 17	10.41±1.26	8.08±1.42
NM_008471	*Krt1–19*	keratin 19	2.71±0.70	3.92±0.33
NM_010661	*Krt12*	keratin 12	0.26±0.09	0.08±0.03
NM_033373	*Krt1–23*	keratin 23	2.02±0.51	2.12±0.28
NM_008475	*Krt2–4*	keratin 4	4.76±0.73	4.49±0.17
NM_033073	*Krt2–7*	keratin 7	4.49±1.41	6.02±0.82
**Proteinase inhibitors**				
XM_138237	*Serpina3f*	serine (or cysteine) peptidase inhibitor, clade A, member 3F	9.87±3.06	7.78±0.52
NM_009252	*Serpina3n*	serine (or cysteine) peptidase inhibitor, clade A, member 3N	17.36±3.33	10.30±2.67
NM_009126	*Serpinb3a*	serine (or cysteine) peptidase inhibitor, clade B, member 3A	18.78±14.65	17.32±3.41
NM_201363	*Serpinb3c*	serine (or cysteine) peptidase inhibitor, clade B, member 3C	20.51±11.56	27.78±5.39
NM_201376	*Serpinb3d*	serine (or cysteine) peptidase inhibitor, clade B, member 3D	24.03±18.60	38.29±12.10
NM_011454	*Serpinb6b*	serine (or cysteine) peptidase inhibitor, clade B, member 6b	5.28±1.79	7.55±0.79
NM_009255	*Serpine2*	serine (or cysteine) peptidase inhibitor, clade E, member 2	3.16±0.24	4.28±0.36
NM_009825	*Serpinh1*	serine (or cysteine) peptidase inhibitor, clade H, member 1	2.09±0.43	3.25±0.63
**Unknown or predicted genes**				
XM_135671	Predicted	PHD finger protein 11	0.46±0.12	0.46±0.10
NM_183249	Predicted	RIKEN cDNA 1100001G20 gene	6.73±2.53	6.55±0.85
XM_128979	Predicted	RIKEN cDNA C330008K14 gene	5.99±1.76	4.15±0.81
NM_175417	Predicted	RIKEN cDNA 9530008L14 gene	0.30±0.04	0.10±0.01
NM_173421	Predicted	cDNA sequence BC030476	0.34±0.06	0.27±0.03
NM_029733	Predicted	RIKEN cDNA 2010005H15 gene	3.72±0.56	2.21±0.40
NM_028166	Predicted	RIKEN cDNA 1600014C10 gene	0.31±0.05	0.38±0.04
NM_027171	Predicted	RIKEN cDNA 2310057J16 gene	0.42±0.15	0.29±0.10
XM_489536	Predicted	RIKEN cDNA 6430590I03 gene	0.33±0.03	0.34±0.07
NM_134133	Predicted	RIKEN cDNA 2010002N04 gene	9.75±1.85	6.34±0.66
NM_001001332	Predicted	cDNA sequence BC1179090	6.73±0.98	2.99±0.35
NM_026412	Predicted	DNA segment, Chr 2, ERATO Doi 750	2.76±0.67	2.21±1.00
XM_486478	Predicted	ferritin light-chain 1	2.15±0.34	2.07±0.12
NM_001082547	Predicted	Gm 5483	11.52±1.39	4.95±0.51

Microarray analysis has been used successfully in many studies to screen for potential key molecules during a specific process, such as infectious keratitis [26-28]. Aiming at providing a panorama of gene changes during the fast-growing phase of CorNV, this paper does not try to provide experimental confirmation of any proposed genes. However, we had been successful in identifying certain potential targets for manipulating CorNV based on our discovery using microarray profiling. For example, *S100A8* and *S100A9* were first observed in this study (Table 3). Considering that *S100A* family members are important for neutrophil functions, later of which are found important in CorNV development [29], we were able to design experiments and confirm that depleting *S100A8* inhibited S-CorNV [10]. We also previously reported that the so-called lens crystallins, for example, the α-, β-, and γ-crystallins, were physiologically expressed in murine corneas [20]. On the other hand, αB-crystallin has been reported to act as a chaperone for *VEGFA* in angiogenesis [30] and to promote tumor angiogenesis by increasing vascular survival during tube morphogenesis [31]. Since the current study showed that they were significantly upregulated during CorNV, it will be worth investigating the exact role of lens crystallins in CorNV, such as by investigating lens crystallin-deficient animals.

Some genes in the jointly upregulated “immune response” GO terms deserve special attention. For example, the complement pathway, especially the alternative activation pathway, has been proven to be involved in several angiogenic conditions, including ocular neovascularization [32-34], the most famous example being the involvement of complement factor H in the pathogenesis of age-related macular degeneration [35]. The fact that all three types of *C1q* subunits (α, β, and either γ or C) were upregulated in a similar pattern (Table 4) may suggest that complement pathways were involved in the pathogenesis of CorNV. Besides, C-type lectins, including domain family 4, member d (*Clecsf8*) and member n (*Clecsf10*; Table 4), have been proven to be involved in the recognition of pathogens by macrophages . The significance of the upregulation of these genes, as well as of those of two Fc genes (Fc receptor, IgE, high affinity I, gamma polypeptide [*Fcer1g*] and Fc receptor, IgG, low affinity III [*Fcgr3*]) in CorNV might be due to the infiltration of various inflammatory cells. Similarly, the study of some genes in other groups, such as semaphorin, 5A (*Sema5a*) under the “blood vessel development” GO term (Table 7) will also be of interest. Sema5a was recently reported to promote angiogenesis by increasing the proliferation and migration of endothelial cells . However, we have no explanation as to why this gene was downregulated in such CorNV models. Obviously, the genes that are concertedly upregulated or downregulated in both models might suggest common pathways for the pathogenesis of these two models. However, the genes that show differential changes in these two models also deserve attention since they might determine the differential presentation of these two models. Furthermore, this study also provided clues to dissecting the functions of the “predicted genes” that were up- or downregulated.

In summary, we compared the changes of genes expression in two commonly used CorNV models and found that while significant differences existed at the levels of gross presentation and histology, some gene-expression change patterns were shared by these two CorNV models. Further studies are needed to help define the role of some promising genes in CorNV, thereby supplementing our current understanding about the pathogenesis of neovascularization diseases. Taking a step further, such studies might reveal new targets that could be used for manipulation of diseases accompanied with CorNV.
